# The Role of Physical Fitness in Emotional Well-Being and Distress during Pregnancy: The GESTAFIT Project

**DOI:** 10.3390/healthcare12171692

**Published:** 2024-08-25

**Authors:** Nuria Marín-Jiménez, Marta Flor-Alemany, Laura Baena-García, Pablo Corres, Cristina Molina-Hidalgo, Virginia A. Aparicio

**Affiliations:** 1 Department of Physical Education and Sports, Faculty of Sport Sciences, University of Granada, 18071 Granada, Spain; nmjimenez@ual.es; 2 Sport and Health University Research Institute (iMUDS), University of Granada, 18007 Granada, Spain; floralemany@ugr.es (M.F.-A.); cristina.molinahidalgo@adventhealth.com (C.M.-H.); virginiaparicio@ugr.es (V.A.A.); 3 GALENO Research Group, Department of Physical Education, Faculty of Education Sciences, University of Cádiz, 11510 Puerto Real, Spain; 4 Biomedical Research and Innovation Institute of Cádiz (INiBICA) Research Unit, 11009 Cádiz, Spain; 5 Department of Physiology, University of Granada, 18011 Granada, Spain; 6 Institute of Nutrition and Food Technology, University of Granada, 18003 Granada, Spain; 7 Department of Nursing, Faculty of Health Sciences, University of Granada, 18071 Granada, Spain; 8 Biosanitary Research Institute, IBS, University of Granada, 18012 Granada, Spain; 9 Department of Physical Education and Sport, Faculty of Education and Sport, Physical Activity and Sport Sciences Section, University of the Basque Country (UPV/EHU), 01006 Vitoria-Gasteiz, Spain; pablo.corres@ehu.eus; 10 AdventHealth Research Institute, Neuroscience Institute, Orlando, FL 32803, USA

**Keywords:** pregnancy/gestation, fitness, positive affect, emotional intelligence, resilience, emotional distress

## Abstract

Pregnancy involves various physiological, physical, and social changes that can impact the mental health of the woman, causing her to have a stressful experience. Physical fitness (PF) is postulated as a powerful marker of health in this population. Therefore, this longitudinal study examined the association of PF with maternal emotional well-being and ill-being outcomes at 16th and 34th gestational weeks (g.w.) in a sample of 158 pregnant women (32.9 ± 4.7 years old). Self-reported PF was assessed with the valid and feasible International Fitness Scale [i.e., overall PF, cardiorespiratory fitness (CRF), muscular strength, speed–agility, and flexibility]; positive and negative affect, emotional intelligence, and resilience were measured using validated questionnaires specifically designed for this purpose. The results showed that women with greater overall PF and its components showed higher positive affect and lower negative affect (all, *p* < 0.05); greater emotional intelligence (all, *p* < 0.05); and greater resilience (all, *p* < 0.05), with similar results both in the 16th and the 34th g.w. These findings underscore the pivotal role of PF in promoting emotional health and resilience during pregnancy, thereby highlighting the need for integrating PF enhancement strategies in prenatal care programs.

## 1. Introduction

Pregnancy is a dynamic period of growth and development, presenting both physical and psychological challenges to expectant mothers [1]. As the fetus’s neurodevelopment unfolds, maternal well-being becomes crucial since the maternal environment, including nutrition, lifestyle, and mental health, can significantly impact fetal development [2]. Thus, it is evident that maternal well-being not only is important for the mother herself but also plays a pivotal role in the health and development of the fetus.

Anxiety and depression are the most common mental disorders during pregnancy, affecting up to 36% of women [3], increasing especially in the third trimester of pregnancy [3]. In this sense, achieving optimal mental health during pregnancy seems particularly relevant since psychological ill-being (i.e., negative affect, understood as emotional events such as sadness, loneliness, anger, lack of motivation, and lack of concentration, which can induce anxiety, depression, and stress) implies greater emotional instability [4]. Moreover, it may increase the risk of deleterious effects on materno-fetal health, such as low birth weight, preterm birth or miscarriage [5,6,7], and physiological development of the offspring [8]. On the other hand, well-being, which includes the construct of positive affect (i.e., the experience of pleasurable emotions, such as happiness, joy, excitement, enthusiasm, calm, and contentment) [9], might positively impact women’s own health and fetal development [10]. Therefore, both low levels of psychological distress and high levels of emotional well-being must be considered to achieve optimal mental health during pregnancy. However, there is still limited scientific evidence regarding the impact of well-being or positive affect on pregnant women [8,11,12].

Likewise, emotional intelligence, which encompasses emotional attention, clarity, and repair [13], plays a crucial role during pregnancy. Emotional attention refers to how much attention individuals pay to their inner feelings and emotional states [13]. Emotional clarity, which is the ability to understand and discriminate among feelings in oneself, aids in handling negative states and reducing distress [13]. Emotional repair is the ability to regulate moods and transform negative feelings into positive behaviors, promoting a positive birth experience and peaceful mother–infant communication [13]. Thus, it has been linked to various aspects of mental health, caregiving, and developmental correlates throughout the perinatal period [14]. Therefore, understanding how emotional intelligence influences a pregnant woman’s experience can guide interventions aimed at promoting mental health during this critical period. Moreover, resilience during pregnancy plays a crucial role in the overall health and well-being of both the mother and the developing fetus. It is the ability to adapt and recover from stressors or adversities, which is particularly important during pregnancy due to the physical and emotional changes that occur [15]. High resilience has been associated with lower levels of prenatal stress and anxiety, which can have significant impacts on fetal development and birth outcomes [16]. Furthermore, resilience can also influence postnatal outcomes, such as reducing the risk of postpartum depression and promoting positive parenting behaviors [17]. Therefore, fostering resilience during pregnancy is of paramount importance for maternal and child health.

In this context, maintaining physical fitness (PF) during pregnancy contributes to overall well-being. It is noteworthy that exercise enhances PF [18], which has been positioned as a powerful health marker in different populations [19,20], including pregnant women and their infants [21]. Indeed, self-reported PF, through validated and widely used scales such as the International Fitness Scale (IFIS), has been shown as a useful and feasible tool to evaluate PF during pregnancy, especially in clinical settings [22].

By integrating exercise interventions with physical fitness components, healthcare providers can empower pregnant women to optimize their health and that of their offspring. However, despite the arising evidence of the positive association of PF levels with pregnancy-related symptoms/outcomes [23,24,25], labor and birth outcomes [26,27], and improved health-related quality of life [22], no previous studies have investigated its association with maternal emotional well-being and emotional distress. Consequently, the aim of the present study was to explore the association of PF with emotional well-being and emotional distress along the pregnancy course [i.e., 16th and 34th gestational weeks (g.w.)].

## 2. Materials and Methods

### 2.1. Study Design and Participants

This longitudinal study presents secondary analyses from the GEStation and FITness (GESTAFIT) project (registration number: NCT02582567) [28]. The study design and complete methodology together with the inclusion–exclusion criteria and procedures were previously published elsewhere [28]. In summary, the inclusion criteria consisted of healthy women aged 25 to 40 years with a normal pregnancy who provided informed consent. The exclusion criteria encompassed high-risk obstetric pregnancies, fetal malformations, and maternal malnutrition, among other conditions (see Supplementary Table S1).

The sample size required for the GESTAFIT Project was calculated solely for the primary outcomes, which included maternal weight gain and maternal/neonatal glycemic profiles [28].

The GESTAFIT project involved a concurrent exercise intervention combining aerobic and resistance training [the exercise intervention was performed in three groups of about nine participants each, 3 days per week (60 min per session)], which was implemented from the 17th g.w. until birth (~40 weeks).

Briefly, the research team recruited participants during the 11th to 13th g.w., coinciding with their initial gynecologist check-up at the “San Cecilio” University Hospital in Granada, Spain. Prior to participation, all interested individuals received detailed information about this study’s objectives and procedures. Subsequently, each participant provided written informed consent. To adhere to ethical standards, we followed the procedures outlined in the Declaration of Helsinki.

Following recruitment during their initial hospital visit, participants were invited to participate in this study at the Sport and Health University Research Institute—iMUDS—in Granada, Spain. Both the assessments and the exercise program were conducted at this research center.

All assessments occurred at two time points: the 16th (±2 weeks) and 34th g.w. (±2 weeks). A total of 158 Spanish pregnant women (32.9 ± 4.6 years old) were recruited in two waves, for feasibility reasons, between November 2015 and March 2017.

### 2.2. Procedures

The evaluation procedures were conducted on two separate days. The initial assessment occurred around the 16th g.w. (±2 weeks), during which participants completed a handwritten self-reported questionnaire on sociodemographic and clinical data, and body composition was also measured. The second assessment was conducted around the 34th g.w. (±2 weeks), where height and weight were measured again, and participants completed self-reported assessments of PF, emotional well-being, and emotional distress, following instructions provided by the research team.

Blood pressure and resting heart rate were recorded prior to the commencement of each evaluation to ensure that the participants were in proper health to perform the physical tests.

### 2.3. Measurements

#### 2.3.1. Sociodemographic and Clinical Data

Sociodemographic data, encompassing variables such as age, number of children, history of abortions, cohabitation status, educational level, and employment status, were evaluated through a self-reported survey (see Table S1).

The research team was present at all times for any explanations or instructions required by the participants.

#### 2.3.2. Anthropometry and Body Composition

Prepregnancy body weight was self-reported at the 11–13th g.w. Body weight and height at the 16th and 34th g.w. were assessed using a scale (InBody R20; Biospace, Seoul, Republic of Korea) and a stadiometer (Seca 22, Hamburg, Germany), respectively. Gestational weight gain (kg) was calculated as weight in each evaluation minus prepregnancy weight (i.e., weight at the 16th g.w. minus prepregnancy weight and weight at the 34th g.w. minus prepregnancy weight).

Measurements were performed by trained evaluators, and all measurements were collected with bare feet, in light sports clothing, and with a 3 h fast at the same time on each assessment day (i.e., morning or afternoon).

#### 2.3.3. Self-Reported Physical Fitness

Self-reported PF was evaluated using the IFIS [29]. The IFIS consists of five Likert-scale questions that assess participants’ perceived overall PF, cardiorespiratory fitness (CRF), muscular strength, speed–agility, and flexibility. Each question corresponds to a range from 1 to 5, with descriptors such as “very poor,” “poor,” “average,” “good,” and “very good.” Higher scores on the IFIS indicate greater self-reported PF. This questionnaire has been previously validated and has been used in studies involving pregnant populations [23,24,25,26], and it has shown good reliability in different populations [30,31,32]. It can be completed in 1–5 min.

#### 2.3.4. Positive and Negative Affects

The Spanish adaptation of the Positive and Negative Affect Schedule (PANAS-S) [33,34] was used. This is a 20-item valid questionnaire widely employed to measure emotional well-being and emotional distress. The PANAS-S assesses relatively short-term fluctuations in mood (“how do you feel right now”). The questionnaire includes two subscales, Positive Affect and Negative Affect, each of which consists of ten items that express affects such as “active”, “nervous”, or “satisfied.” This questionnaire must be answered on a 5-point Likert scale, from 1 = “very slightly or not at all” to 5 = “extremely”. The score ranges from 10 to 50 for both subscales (Positive Affect and Negative Affect). Higher positive scores reflect greater affective well-being, and higher negative scores show greater emotional distress. Its reliability has been found to be good in different populations (Cronbach’s alpha between 0.86 and 0.90) [35,36].

#### 2.3.5. Emotional Intelligence

The valid and reliable (Cronbach’s alpha 0.85) Spanish-adapted version of the Trait Meta-Mood Scale (TMMS) was utilized to evaluate emotional intelligence [37]. Specifically, it assessed emotional attention, emotional clarity, and emotional regulation. The modified Spanish TMMS consists of three subscales, each comprising eight items rated on a 5-point Likert scale (ranging from 1 to 5). The total scores on the TMMS range from 8 to 40, with higher scores indicating greater emotional attention, clarity, and regulation. 

#### 2.3.6. Resilience

The valid and reliable (Cronbach’s alpha 0.85) Connor–Davidson Resilience Scale (CD-RISC) was utilized to evaluate resilience, defined as an individual’s capacity to prosper in the face of adversity [38,39]. The CD-RISC is composed of 10 elements, each rated on a scale from 0 to 4. Consequently, the cumulative score can range from 0 to 40, with elevated scores signifying enhanced resilience.

### 2.4. Statistical Analyses

Descriptive statistics [mean and standard deviation for quantitative variables and number of women (%) for categorical variables] were employed to describe baseline characteristics of the study participants. Linear regression analyses were performed to explore the association of overall self-reported PF, CRF, muscular strength, speed–agility, and flexibility with emotional well-being and emotional distress at the 16th and 34th g.w. Two models were analyzed. Model I included age and gestational weight gain at the 16th or 34th g.w. as covariates. Model II was additionally adjusted for educational level, working status, and living with a partner. These variables were included since they have previously been shown to be potential determinants of health during pregnancy [40]. At the 34th g.w., Model II was additionally adjusted for exercise intervention in order to correct the possible effect of the exercise program conducted within the GESTAFIT project on emotional well-being and emotional distress.

All analyses were performed using the Statistical Package for Social Sciences (IBM SPSS Statistics for Windows, version 22.0, Armonk, NY, USA), and level of significance was set at *p* ≤ 0.05.

## 3. Results

**Figure 1:**
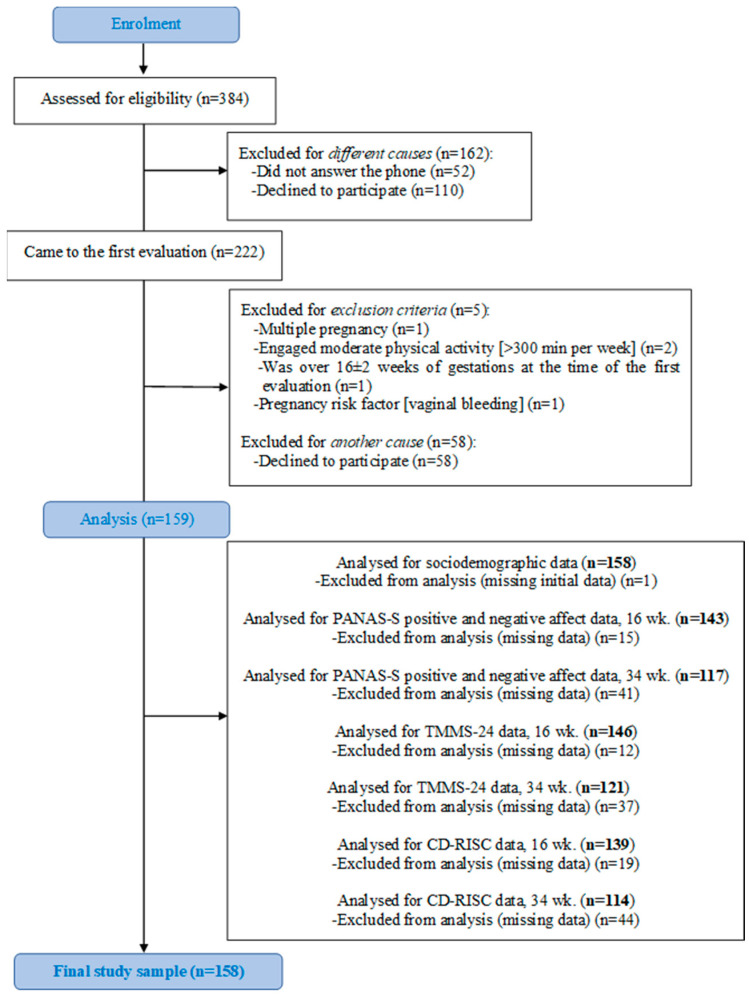
Flow diagram of study participants.

The present study comprised a total of 158 pregnant women with valid baseline data (i.e., 16th g.w.). Nonetheless, there was a loss of data in some outcomes, due to some participants not attending the second evaluation (at the 34th g.w.) or not returning all the questionnaires duly filled (see Figure 1).

The sociodemographic, anthropometric, and clinical characteristics of the participants are presented in Table 1. Women’s gestational weight gain at the 16th and 34th g.w. were 2.1 ± 2.8 kg and 10.6 ± 5.0 kg, respectively. Pregnant women showed an average level of overall self-reported PF and all its components through the pregnancy course. Almost 90% of the pregnant women had completed higher studies than primary or high school, and around 70% of them were employed at baseline. Positive affect values were slightly higher at 16th g.w. than at 34th g.w., while negative affect values were slightly higher at 34th g.w. than at 16th g.w. Emotional intelligence dimensions remained unchanged throughout pregnancy, with high values (~30). The same high values were found for resilience throughout pregnancy (~30).

Associations of overall self-reported PF and its components with emotional well-being and emotional distress at the 16th g.w. are shown in Table 2. In Model II, women who reported greater overall self-reported PF, CRF, muscular strength, and speed–agility showed greater positive affect (β ranging from 0.194 to 0.299; all, *p* < 0.05); greater overall self-reported PF, CRF, speed–agility, and flexibility were associated with greater emotional clarity (β ranging from 0.183 to 0.282; all, *p* < 0.05). No associations were found between PF and negative affect and emotional attention, emotional repair, or resilience. In Model I, the results remained the same.

Associations of self-reported overall PF and its components with emotional well-being and emotional distress at the 34th g.w. are shown in Table 3. In Model II, women who reported greater overall self-reported PF, CRF, muscular strength, and speed–agility showed greater positive affect (β ranging from 0.227 to 0.299; all, *p* < 0.05); greater overall self-reported PF, CRF, muscular strength, and speed–agility were associated with lower negative affect (β ranging from −0.217 to −0.241; all *p* < 0.05); greater overall self-reported PF was associated with greater emotional clarity (β = 0.201, *p* = 0.049); and greater overall self-reported PF, CRF, muscular strength, and flexibility were associated with greater resilience (β ranging from 0.196 to 0.238; all *p* < 0.05). In Model I, the results remained the same.

## 4. Discussion

In the current study, we examined, for the first time, the association of self-reported PF with emotional well-being (e.g., positive affect, emotional intelligence, and resilience) and emotional distress (e.g., negative affect) along the pregnancy course. We found that greater self-reported PF was associated with greater emotional well-being and less emotional distress during pregnancy (e.g., 16th and 34th g.w.). Specifically, greater self-reported PF during early pregnancy (i.e., 16th g.w.) was associated with positive mood and emotional clarity along the pregnancy course. Likewise, greater self-reported PF in late pregnancy (i.e., 34th g.w.) was associated with reduced negative differences in affectivity and resilience during this period.

As far as we know, the fact that overall self-reported PF is associated with greater emotional well-being and lower emotional distress during pregnancy has public health and clinical implications, since well-being during pregnancy may be compromised due to pregnancy-related physical and psychological changes [1]. 

Although we found that greater self-reported PF levels are linked to greater positive affect and lower negative affect during pregnancy, the associations found for greater PF levels with better positive affect remain significant during the pregnancy course (β ranging from 0.194 to 0.299; all, *p* < 0.05), and the associations of PF levels with lower negative affect are especially relevant during late pregnancy (i.e., at 34th g.w.; β ranging from −0.217 to −0.241; all, *p* < 0.05). Somehow, this fact may be related to typical psychological symptoms such as higher rates of anxiety and depression during late pregnancy [3] and some possible fears and worries related with the term of the pregnancy (such as fear associated with complications during labor or to give birth in itself) [3].

In the context of emotional intelligence, the influence of self-reported PF during pregnancy has not been previously investigated. Our findings suggest that greater PF levels are positively associated with emotional clarity, particularly during early pregnancy (i.e., at 16th g.w.; β ranging from 0.183 to 0.282; all, *p* < 0.05). This suggests that women with greater PF levels may have higher self-awareness and deeper connection with their emotions during pregnancy, which has been linked to handling negative states and reducing distress [41]. In this sense, our research group previously explored the relationship between physical activity, sedentary behavior, and objectively measured PF with emotional intelligence [42], finding that only flexibility was associated with emotional repair during the early stages of pregnancy using the same sample [42]. Although small relationships between higher physical activity and higher emotional intelligence, especially in attention and repair, were previously reported [41], this study included undergraduate female and male students in its sample. Additionally, it should be noted that our sample scored better that those reported by the female sample in the above-mentioned study, and the male participants were more physically active than their female pairs, which may be somehow associated with the observed results.

Likewise, our results showed that greater self-reported PF was positively associated with greater resilience during late pregnancy (i.e., at 34th g.w.; β ranging from 0.196 to 0.238; all *p* < 0.05). These finding are especially relevant since resilience has the potential to counteract the negative impact of stress and can be a protective factor against mental health problems [17]. Moreover, higher resilience can protect women from vulnerability and perceived stress, potentially preventing complications and contributing to a positive experience during pregnancy [17], since high levels of maternal pregnancy stress are associated with an increased risk for adverse birth outcomes as well as anxiety and depression symptoms during and following pregnancy [16]. Similar results have been reported in a sample of adults (aged ~27 years) including males and females [43]. However, no previous studies have explored the relationship between self-reported PF and resilience during pregnancy.

Despite the lack of studies investigating the relationship of self-reported PF levels with maternal emotional well-being and emotional distress during pregnancy, some mechanisms have been previously proposed which may explain our findings. First, greater PF levels may decrease physiological and metabolic reactivity to stressful events, optimizing hormonal stress responses and preventing many chronic diseases. In this sense, exercise may play a key role in the regulation of stress hormones, such as cortisol, via the hypothalamus–pituitary–adrenal axis and the autonomic nervous system [44]. Indeed, elevated cortisol levels, present in physiological states of high physical or mental stress, are a potential biological mechanism leading to health complications in pregnant women and fetal adverse outcomes (such as premature births or low APGAR scores) [45]. Second, exercise also releases β-endorphins that produce an analgesic effect, promoting positive mood and a sense of well-being [46]. Additionally, greater PF levels through exercise enhance growth factor expression and neural plasticity, contributing to improvements in mood and cognition [44]; the release of myokines from skeletal muscles induces neuroprotection (increasing expression of brain-derived neurotrophic factor), demonstrating anxiolytic and antidepressant effects [47], as well as the mediating role of exercise by a decrease in the number of microglia and the suppression of neuroinflammation in the hippocampus [48]. Finally, greater overall PF levels promote improved social factors, sociability [49], self-esteem, self-efficacy, distraction [50], motivation [1], and better quality of life [51]. In conclusion, women with high/adequate PF before pregnancy or those reaching greater overall PF levels during pregnancy show greater psychological well-being [52], and this is an interesting and safe option in the prevention and treatment of maternal distress. Future longitudinal studies analyzing the individual association of PF components with emotional well-being and emotional distress during pregnancy are warranted to confirm our results.

### Limitations and Strengths

There are a few limitations that should be acknowledged. Although analyses were performed controlling for potential confounders (i.e., educational level, working status, and living with a partner), it is possible that there exist other unstudied confounders that affect emotional well-being and distress. In addition, our sample only included Caucasian women with a high educational level, so our results cannot be extrapolated to other types of populations. Nevertheless, the current study has a number of strengths. First, we analyzed outcomes from the early second trimester of pregnancy to late pregnancy, providing a wide overview of the gestational period. Second, although PF levels were determined using self-reported approaches, the IFIS is a tool validated in a pregnant population, largely used in epidemiological studies [22]. In fact, while our group had previously established its association with quality of life, we had yet to delve into more emotional spheres, such as those presented in the current study.

## 5. Conclusions

Our study provides compelling evidence that greater self-reported PF is associated with greater emotional well-being and less emotional distress during pregnancy. Notably, greater self-reported PF during early pregnancy appears to be related to positive affect and emotional clarity throughout the gestational period. Furthermore, an increased PF in late pregnancy is particularly crucial, not only for fostering positive mood but also for mitigating negative affect disparities and bolstering resilience during this critical phase. These findings underscore the potential positive role of PF on emotional health and resilience during pregnancy, thereby highlighting the need for integrating PF enhancement strategies in prenatal care programs. Future research should aim to further elucidate the underlying mechanisms and potential interventions to optimize these outcomes.

## Figures and Tables

**Table 1:** Sociodemographic characteristics, anthropometric measures, and self-reported physical fitness levels of the participants.

Maternal Outcomes	16th Gestational Week		34th Gestational Week	
	n (Mean ± SD)			
Age (years)	158 (33.0 ± 4.7)			
Weight previous to pregnancy (kg)	145 (65.1 ± 12.3)			
Gestational weight gain (prepregnancy-16th g.w.)	143 (2.1 ± 2.8)			
Gestational weight gain (prepregnancy-34th g.w.)	118 (10.6 ± 5.0)			
	n		n (%)	
Living with a partner (yes)	158		154 (97.5)	
Educational level	158			
Primary or high school			18 (11.4)	
Specialized training			46 (29.1)	
University degree			94 (59.5)	
Working status	158			
Homework/unemployed student			48 (30.4)	
Part-time employed/student			41 (25.9)	
Full-time employed			69 (43.7)	
	n	Mean ± SD	n	Mean ± SD
Self-Reported Physical Fitness (0–5)	157	3.2 ± 0.8	117	3.3 ± 0.8
Overall physical fitness		2.5 ± 0.9		2.6 ± 0.8
Cardiorespiratory fitness		3.1 ± 0.8		3.3 ± 0.7
Muscular strength		3.1 ± 0.8		3.0 ± 0.8
Speed–agility		3.1 ± 0.8		3.1 ± 1.0
Flexibility		3.2 ± 0.8		3.3 ± 0.8
PANAS-S	143		117	
Positive affect (10–50) ^a^		34.1 ± 6.7		32.9 ± 7.6
Negative affect (10–50) ^b^		17.6 ± 7.1		18.6 ± 6.9
TMMS-24				
Attention dimension (TMMS-A, 8–40) ^c^	146	25.4 ± 6.2	121	25.5 ± 6.0
Clarity dimension (TMMS-C, 8–40) ^d^		30.51 ± 4.9		30.2 ± 5.5
Repair dimension (TMMS-R, 8–40) ^e^		29.8 ± 5.2		29.9 ± 5.2
CD-RISC (0–40) ^f^	139	30.1 ± 5.5	114	29.9 ± 5.3

SD, standard deviation; PANAS-S, Positive and Negative Affect Schedule–State; TMMS-24, Trait Meta-Mood Scale 24 items; CD-RISC, Connor–Davidson Resilience Scale. ^a^ Higher scores reflect greater affective emotional health/experience. ^b^ Higher scores reflect greater emotional distress. ^c^ Higher scores reflect greater attention. ^d^ Higher scores reflect greater clarity. ^e^ Higher scores reflect greater regulation. ^f^ Higher scores indicate greater resilience.

**Table 2:** Association of self-reported overall physical fitness and its components with emotional well-being and distress at the 16th g.w.

		Model I						Model II				
	β	B	95% CI		Adj. R^2^	*p*	β	B	95% CI		Adj. R^2^	*p*
			Lower	Upper					Lower	Upper		
	PANAS-S Positive Affect (n = 127)											
Overall physical fitness	0.215	1.873	0.323	3.423	0.047	**0.018**	0.212	1.851	0.284	3.417	0.033	**0.021**
Cardiorespiratory fitness	0.206	1.488	0.210	2.765	0.044	**0.023**	0.194	1.400	0.075	2.726	0.024	**0.039**
Muscular strength	0.294	2.560	1.075	4.045	0.089	**0.001**	0.299	2.600	1.097	4.104	0.079	**0.001**
Speed–agility	0.264	2.163	0.757	3.569	0.073	**0.003**	0.285	2.332	0.908	3.757	0.070	**0.002**
Flexibility	0.065	0.425	−0.739	1.589	0.005	0.471	0.067	0.437	−0.740	1.615	0.010	0.463
	PANAS-S Negative Affect (n = 127)											
Overall physical fitness	−0.137	−1.279	−2.974	0.415	0.008	0.138	−0.139	−1.297	−2.998	0.404	0.006	0.134
Cardiorespiratory fitness	−0.145	−1.121	−2.514	0.272	0.010	0.114	−0.135	−1.042	−2.472	0.389	0.006	0.152
Muscular strength	−0.072	−0.671	−2.342	1.000	0.005	0.428	−0.084	−0.782	−2.479	0.916	0.019	0.364
Speed–agility	−0.129	−1.135	−2.694	0.424	0.007	0.152	−0.140	−1.226	−2.811	0.360	0.004	0.128
Flexibility	−0.098	−0.685	−1.936	0.567	0.002	0.281	−0.091	−0.636	−1.908	0.636	0.017	0.324
	TMMS-A (n = 122)											
Overall physical fitness	0.045	0.359	−1.121	1.840	0.001	0.632	0.033	0.265	−1.203	1.733	0.028	0.721
Cardiorespiratory fitness	−0.015	−0.102	−1.325	1.122	0.002	0.869	−0.002	−0.014	−1.247	1.219	0.027	0.982
Muscular strength	−0.006	−0.049	−1.553	1.454	0.002	0.948	−0.006	−0.052	−1.538	1.435	0.027	0.945
Speed–agility	−0.027	−0.203	−1.571	1.166	0.002	0.770	−0.044	−0.332	−1.696	1.031	0.029	0.630
Flexibility	0.028	0.170	−0.913	1.252	0.001	0.757	0.030	0.177	−0.895	1.249	0.030	0.744
	TMMS-C (n = 122)											
Overall physical fitness	0.289	1.869	0.717	3.020	0.064	**0.002**	0.282	1.822	0.673	2.971	0.079	**0.002**
Cardiorespiratory fitness	0.230	1.233	0.267	2.199	0.035	**0.013**	0.207	1.110	0.127	2.094	0.042	**0.027**
Muscular strength	0.128	0.853	−0.355	2.061	0.001	0.165	0.141	0.941	−0.258	2.140	0.021	0.123
Speed–agility	0.193	1.172	0.084	2.261	0.020	**0.035**	0.201	1.221	0.132	2.311	0.041	**0.028**
Flexibility	0.196	0.947	0.083	1.811	0.021	**0.032**	0.183	0.883	0.021	1.746	0.035	**0.021**
	TMMS-R (n = 122)											
Overall physical fitness	0.157	1.055	−0.175	2.285	0.010	0.092	0.142	0.953	−0.270	2.176	0.033	0.126
Cardiorespiratory fitness	0.140	0.778	−0.241	1.796	0.005	0.133	0.096	0.534	0.499	1.556	0.022	0.308
Muscular strength	0.092	0.641	−0.617	1.900	0.006	0.315	0.096	0.664	−0.580	1.909	0.022	0.293
Speed–agility	0.108	0.677	−0.467	1.821	0.003	0.243	0.136	0.853	−0.284	1.991	0.031	0.140
Flexibility	0.098	0.492	−0.418	1.402	0.004	0.286	0.097	0.487	−0.416	1.390	0.021	0.287
	CD-RISC (n = 125)											
Overall physical fitness	0.076	0.548	−0.789	1.885	0.018	0.419	0.074	0.528	−0.824	1.879	0.031	0.441
Cardiorespiratory fitness	0.072	0.427	−0.674	1.528	0.018	0.444	0.062	0.369	−0.772	1.510	0.033	0.523
Muscular strength	0.148	1.057	−0.234	2.347	0.001	0.108	0.144	1.027	−0.282	2.335	0.015	0.123
Speed–agility	0.098	0.653	−0.556	1.863	0.014	0.287	0.118	0.786	−0.448	2.019	0.022	0.210
Flexibility	0.053	0.286	−0.688	1.261	0.020	0.562	0.061	0.329	−0.656	1.313	0.034	0.510

PANAS-S, Positive and Negative Affect Schedule–State; items; TMMS-A, Trait Meta-Mood Attention dimension; TMMS-C, Trait Meta-Mood Clarity dimension; TMMS-R, Trait Meta-Mood Repair dimension; CD-RISC, Connor–Davidson Resilience Scale. β, standardized regression coefficient; B, nonstandardized regression coefficient; CI, confidence interval; Adj. R^2^, adjusted coefficient of determination. Statistically significant associations (*p* < 0.05) are highlighted in bold. Model I adjusted for age and gestational weight gain at 16th gestational week. Model II additionally adjusted for educational level, working status, and living with a partner.

**Table 3:** Association of self-reported overall physical fitness and its components with emotional well-being and distress at the 34th g.w.

		Model I						Model II				
	β	B	95% CI		Adj. R^2^	*p*	β	B	95% CI		Adj. R^2^	*p*
			Lower	Upper					Lower	Upper		
	PANAS-S Positive Affect (n = 107)											
Overall physical fitness	0.255	2.556	0.603	4.509	0.042	**0.011**	0.227	2.268	0.268	4.269	0.035	**0.027**
Cardiorespiratory fitness	0.306	2.962	1.136	4.788	0.073	**0.002**	0.282	2.732	0.867	4.598	0.065	**0.005**
Muscular strength	0.294	2.560	1.075	4.045	0.001	**0.001**	0.299	2.600	1.097	4.104	0.008	**0.001**
Speed–agility	0.264	2.163	0.757	3.569	0.005	**0.003**	0.285	2.332	0.908	3.757	0.011	**0.002**
Flexibility	0.115	0.881	−0.616	2.378	0.008	0.246	0.067	0.437	−0.740	1.615	0.005	0.463
	PANAS-S Negative Affect (n = 107)											
Overall physical fitness	−0.247	−2.232	−4.00	−0.464	0.035	**0.014**	−0.241	−2.173	−4.000	−0.346	0.010	**0.020**
Cardiorespiratory fitness	−0.234	−2.042	−3.726	−0.359	0.031	**0.018**	−0.224	−1.962	−3.698	−0.226	0.005	**0.027**
Muscular strength	−0.217	−2.127	−4.051	−0.203	0.022	**0.031**	−0.217	−2.126	−4.105	−0.146	0.001	**0.036**
Speed–agility	−0.239	−2.253	−4.058	−0.449	0.034	**0.015**	−0.241	−2.272	−4.100	−0.444	0.015	**0.015**
Flexibility	−0.200	−1.387	−2.721	−0.054	0.017	**0.042**	−0.194	−1.347	−2.707	0.013	0.007	0.052
	TMMS-A (n = 109)											
Overall physical fitness	−0.065	−0.500	−1.993	0.992	0.033	0.508	−0.078	−0.600	−2.130	0.930	0.033	0.438
Cardiorespiratory fitness	−0.137	−1.040	−2.457	0.377	0.042	0.149	−0.142	−1.050	−2.482	0.382	0.047	0.149
Muscular strength	−0.095	−0.783	−2.384	0.818	0.037	0.334	−0.121	−1.002	−2.623	0.619	0.041	0.223
Speed–agility	−0.051	−0.409	−1.930	1.113	0.031	0.595	−0.041	−0.326	−1.855	1.204	0.029	0.674
Flexibility	−0.234	−0.040	−1.349	0.881	0.030	0.678	−0.037	−0.217	−1.344	0.909	0.028	0.703
	TMMS-C (n = 122)											
Overall physical fitness	0.232	1.634	0.260	3.007	0.040	**0.020**	0.201	1.414	0.004	2.823	0.038	**0.049**
Cardiorespiratory fitness	0.054	0.373	−0.975	1.721	0.009	0584	0.016	0.113	−1.256	1.482	0.001	0.871
Muscular strength	0.035	0.266	−1.253	1.786	0.011	0.729	0.031	0.239	−1.292	1.771	0.001	0.757
Speed–agility	0.133	0.988	−0.445	2.420	0.006	0.175	0.124	0.917	−0.516	2.349	0.016	0.207
Flexibility	0.057	0.314	−0.749	1.378	0.009	0.559	0.033	0.180	−0.890	1.251	0.001	0.739
	TMMS-R (n = 122)											
Overall physical fitness	0.171	1.731	−0.169	2.489	0.022	0.086	0.131	0.884	−0.469	2.236	0.035	0.198
Cardiorespiratory fitness	0.177	1.163	−0.096	2.421	0.025	0.070	0.145	0.948	−0.325	2.220	0.040	0.143
Muscular strength	0.103	0.751	−0.691	2.193	0.004	0.304	0.113	0.830	−0.613	2.272	0.031	0.257
Speed–agility	0.120	0.847	−0.516	2.210	0.008	0.220	0.108	0.763	−0.589	2.116	0.031	0.265
Flexibility	0.187	0.972	−0.015	1.960	0.029	0.054	0.167	0.869	−0.119	1.856	0.048	0.084
	CD-RISC (n = 125)											
Overall physical fitness	0.260	1.763	0.404	3.122	0.033	**0.011**	0.238	1.612	0.218	3.006	0.022	**0.024**
Cardiorespiratory fitness	0.235	1.537	0.256	2.818	0.024	**0.019**	0.212	1.382	0.073	2.692	0.013	**0.039**
Muscular strength	0.228	1.664	0.194	3.134	0.019	**0.027**	0.218	1.590	0.099	3.082	0.014	**0.037**
Speed–agility	0.145	1.021	−0.380	2.421	0.010	0.151	0.149	1.044	−0.363	2.451	0.009	0.144
Flexibility	0.202	1.065	0.029	2.101	0.010	**0.044**	0.196	1.034	−0.013	2.082	0.008	**0.053**

PANAS-S, Positive and Negative Affect Schedule–State;, Trait Meta-Mood Scale 24 items; TMMS-A, Trait Meta-Mood Attention dimension; TMMS-C, Trait Meta-Mood Clarity dimension; TMMS-R, Trait Meta-Mood Repair dimension; CD-RISC, Connor–Davidson Resilience Scale. β, standardized regression coefficient; B, nonstandardized regression coefficient; CI, confidence interval; Adj. R^2^, adjusted coefficient of determination. Statistically significant associations (*p* < 0.05) are highlighted in bold. Model I adjusted for age and gestational weight gain at 34th gestational week. Model II additionally adjusted for exercise intervention, educational level, working status, and living with a partner.

## Data Availability

The data that support the findings of this study are available from the corresponding author, MFA, upon reasonable request.

## Supplementary Materials

The following supporting information can be downloaded at: https://www.mdpi.com/article/10.3390/healthcare12171692/s1, Supplementary Table S1. Study inclusion and exclusion criteria.
