# Supplementary material for: The Role of Physical Fitness in Emotional Well-Being and Distress during Pregnancy: The GESTAFIT Project
Source: Healthcare (Basel). 2024 Aug 25;12(17):1692. doi: 10.3390/healthcare12171692 (PMC11395653; doi:10.3390/healthcare12171692)
Supplement: Supplementary file 1 [file healthcare-12-01692-s001.zip › healthcare-3121481-supplementary.pdf]

Supplementary Table S1. Study inclusion and exclusion criteria.

Inclusion criteria
- Pregnant women aged 25–40 years old with a normal pregnancy course.
- Answering "no" to all questions on the PARmed-X for pregnancy.
- Being able to walk without assistance.
- Being able to read and write properly.
- Informed consent: being capable of and willing to provide written consent.
*In addition, specific inclusion criteria for data analysis are as follows: pregnancy with single fetus, spontaneous or instrumental vaginal birth, and caesarean without materno-fetal pathology (or any other indication that does not involve materno-fetal risk, such as disproportion, failed induction, no fetal progression, or noncephalic presentation).
Exclusion criteria
- Acute or terminal illness.
- Malnutrition.
- Inability to conduct tests for assessing physical fitness or exercise during pregnancy.
- Underweight.
- Pregnancy risk factors (such as hypertension, type 2 diabetes, etc.).
- Multiple pregnancies.
- Chromosopathy or fetal malformations.
- Uterine growth restriction.
- Fetal death.
- Upper or lower extremity fracture in the past 3 months.
- Presence of neuromuscular disease or drugs affecting neuromuscular function.
- Doing more than 300 minutes of at least moderate physical activity per week.
- Being registered in another exercise program.
- Unwillingness either to complete the study requirements or to be randomized into the control or intervention group.
